# Medical Intelligence

**Published:** 1848

**Authors:** 


### ARTICLE VI.

### MEDICAL INTELLIGENCE.

At the recent commencement of the Rush Medical College, the degree of Doctor of Medicine was conferred on thirty young gentlemen who had complied with the requirements of the institution, and satisfactorily passed the ordeal of examination.

We observe by our excellent exchange, the Southern Medical and Surgical Journal, that the States of South Carolina and Alabama have recently organized State Medical Associations. The physicians of Pennsylvania were to have held a meeting at Lancaster, on the 11th of April, for the same purpose.

We learn by a letter from Dr. A. B. Price, of Boone Grove, Indiana, that he recently attended a patient only two years old, that passed from the bowels six hundred worms, (ascaris lumbricoides) in the course of six weeks. The child was convalescent.

The measles are prevalent in Georgia. Scarletina prevails in different parts of Indiana. There have been quite a number of cases, mostly of a mild form, at Indianapolis, during March and April, but it has nearly, if not entirely, disappeared. No case of the small-pox has occurred here for nearly three months.

A lady in Georgia, while under the influence of ether, suddenly and permanently recovered her hearing.

The medical class of the University of Pennsylvania, to evince their respect for Prof. Chapman, last winter, procured his portrait to be taken in the best style, and presented it to the Wistar Museum.

From accounts received, we have no doubt but the National Medical Association, which convenes in Baltimore tomorrow, will be the largest meeting of physicians ever held in the United States. Dr. Herrick, one of the editors of the Journal, has gone as a delegate from Rush Medical College.

We shall expect, in our next number, to be able to furnish an interesting account of its proceedings.

A case of congenital absence of both eyes in a negro child is reported by Dr. Williman, in the Charleston Medical Journal and Review. The deficiency is simply of the globes.

Prof. Syme, of Edinburgh, succeeds Liston in the chair of surgery, in the London University College Hospital.

The Willoughby Medical College, which was, about a year ago, removed to Columbus, Ohio, has been dispensed with, and its faculty transferred to the Starling Medical College, which Lyne Starling, Esq., of that place, recently endowed by a donation of thirty thousand dollars.

A portion of the Indiana Hospital for the Insane, sufficient for the accommodation of fifty patients, will be ready for their reception by the latter part of July next. The medical Superintendent to be appointed as successor to Dr. Evans, who resigns the place, has not yet been selected by the board of Commissioners.

Dr. Post, of New York, recently relieved a case of violent paroxysmal cough, that came on at night, in which no disease was detected, except redness of the fauces, and slight enlargement of the uvula, by the application of a strong solution of nit. argent. to these parts.

Dr. Thomas T. Hewson, of Philadelphia, for many years one of the surgeons of the Pennsylvania Hospital, and the Philadelphia Alms-House, died on the 19th of February last, aged 75 years. He was, at the time of his death, President of the Philadelphia College of Physicians.

Dr. Jacob Randolph, son-in-law of the late Dr. Physick, of Philadelphia, one of the surgeons of the Pennsylvania Hospital, &c., died recently. E.

